# Integration of the Rac1- and actin-binding properties of Coronin-1C

**DOI:** 10.4161/21541248.2014.992259

**Published:** 2015-04-10

**Authors:** Frances C Tilley, Rosalind C Williamson, Paul R Race, Thomas C Rendall, Mark D Bass

**Affiliations:** 1 School of Biochemistry; University of Bristol; Bristol, UK; 2 Department of Engineering; University of Bristol; Bristol, UK

**Keywords:** actin, Coronin-1C, endocytosis, Rac1, trafficking

## Abstract

The coronin family of actin-binding proteins regulate actin branching by inhibiting Arp2/3. We recently reported 2 interactions that were unique to coronin-1C: binding of a Rac1 inhibitor, RCC2, to the unique linker region and Rac1 itself to the propeller domain in a manner that differs from that proposed for other coronins. Through these interactions coronin-1C redistributes Rac1 from the back of the cell to the leading edge for either activation or sequestration by the associated Rac1-inhibitor, RCC2. Here we investigate the relationship between the Rac1- and actin-binding properties of coronin-1C and find that, although actin appears to be involved in the retrafficking of Rac1, signaling by Rac1 lies upstream of the stress fiber-formation, for which the coronins were originally characterized.

## Abbreviations

ECM: extracellular matrix
PAK: p21-activated kinase
CRIB: Cdc42/Rac-interacting binding domain
GEF: guanine nucleotide exchange factor

## 

Directional and persistent cell migration is essential for a large number of physiological processes in multicellular organisms. In the embryo, complex patterns of cell migration are involved in proper tissue formation. In the adult, the migration of leukocytes into damaged or infected tissue is crucial for an effective immune response, and migration of keratinocytes and dermal fibroblasts are key aspects of wound healing. In addition, the etiology of a wide variety of diseases features aberrant cell migration, ranging from metastases in cancer to autoimmune syndromes.^1^

Motile cells possess the ability to migrate in all directions, and only in response to chemoattractants such as growth factors and extracellular matrix (ECM) proteins do cells polarize.^2,3^ Extracellular stimuli cause the formation of a polarized, branched-actin lamellipodium that is responsible for protrusion at the leading edge of a motile cell.^4^ The current challenge is in understanding how cell polarity is achieved, despite the diffuse distribution of the molecules involved. ECM receptors, such as integrins and syndecan-4, are expressed across the surface of the cell and signal to the cytoskeletal regulator, Rac1, which is also diffusely distributed. The roles of such molecules in directing *in vivo* migration in vertebrates is exemplified by the fibroblast migration defects, and consequently wound healing defects, caused by disrupting syndecan-4 or Rac1 in mice.^5-7^

**Figure 1.**
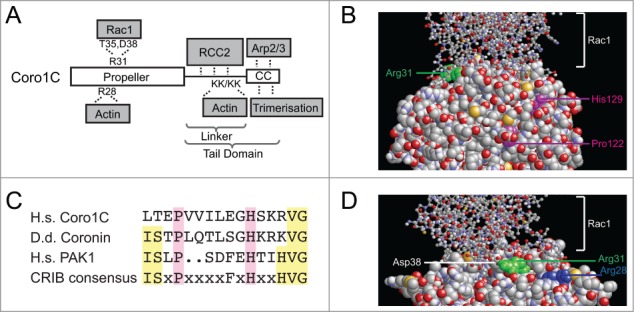
Coronin-1C contains multiple overlapping binding sites. (**A**) Schematic of the domain structure of coronin-1C, indicating the binding sites for the various binding partners and key residues involved. (**B and D**) Structural models of the binding interface between coronin-1C (spacefilling) and GDP-Rac1 (ball and stick). The key Arg31 residue of coronin-1C that mediates Rac1-binding is highlighted in green. The Pro122 and His129 residues that are conserved between human and *Dictyostelium* and mediate binding of Rac1 to *Dictyostelium* coronin are highlighted in magenta. The key Arg28 residue that is conserved between Type I coronins and mediates binding of Rac1 to the propeller is highlighted in blue. (**C**) Sequence alignment comparing the putative CRIB domains of human coronin-1C, *Dictyostelium* coronin and PAK1 with the consensus motif.

However, until recently, mechanisms by which cells distinguish between ECM receptor engagement at the front and sides of the cell have been lacking, as have explanations of how Rac1 is redistributed from the sides to the front of a migrating cell, and how the cytoskeleton feeds back into Rac1 regulation. In a recent paper from our laboratory, we identified the actin-binding protein, coronin-1C, as a key regulator of Rac1 trafficking that was necessary for Rac1 activation at the leading edge.^8^ Coronin-1C is a multi-domain protein, comprising a β-propeller, which may act as a protein scaffold, includes an actin binding site, and is required for membrane localization;^9^ a unique linker region that contains a second actin-binding site;^10^ and a C-terminal coiled-coil which is responsible for formation of homotrimers and binds to both the cell membrane^11^ and the actin-related protein, Arp2/3^12^ (**Fig. 1A**). We identified 2 further features: a binding site for GDP-Rac1 in the propeller domain and an RCC2-binding site in the unique linker region.^8^ The interaction with RCC2 is particularly significant as we also found that RCC2, which associates with adhesion complexes,^13^ acts as a competitive inhibitor of Rac1 activation.^8^ Together, RCC2 and coronin-1C were found to mediate the redistribution and modulate the activation of Rac1, thus focusing Rac1 activation into a single protrusive membrane in response to fibronectin stimulation. It has been proposed that binding of type I coronins to Arp2/3 modulates actin branching in a concentration-dependent manner and may be responsible for focusing regions of membrane protrusion.^9^ The ability of coronin-1C to selectively target Rac1 to similar regions suggests that coronin-1C plays a major role in migration guidance.

To better understand the nature of the coronin-1C/Rac1 interaction, molecular docking was used to generate a model that would allow us to predict key interacting residues. In the absence of a coronin-1C crystal structure, a homology model of this protein was generated from the coronin-1A structure^14^ and used in combination with the available Rac1-GDP crystal structure^15^ in a series of docking simulations. A single high probability Rac1-GDP/coronin-1C complex model was identified (**Fig. 1B**). Binding was predominantly via complementary electrostatics and hydrogen bonding between the face of the coronin-1C propeller and the switch I and switch II regions of Rac1. The key binding site involved the electrostatic interactions of Arg31 of coronin-1C (**Fig. 1B**, green) with the carboxyl oxygens of Asp38 and hydroxyl group of Thr35 of Rac1. The importance of these interactions have been verified by mutagenesis.^8^ Interestingly, it has been reported that *Dictyostelium discoideum* coronin contains a partially conserved Cdc42- Rac1-interactive binding (CRIB) domain that may also mediate interaction with Rac1.^16^ However, although human coronin-1C contains the critical Pro122 and His129 residues, the rest of the consensus motif is more poorly conserved (**Fig. 1C**). The proline and histidine are partially buried (**Fig. 1B**, magenta) and do not overlap with the verified Rac1-binding motif. Thus it appears that the Rac1-binding CRIB motif is not conserved between *Dictyostelium* coronin and human coronin-1C. Furthermore, the Rac1-binding Arg31 is not conserved in human coronin-1A. The different effects of coronin-1C and coronin-1A on Rac1 are manifested at the functional level with coronin-1A delivering Rac1 to the plasma membrane, and coronin-1C facilitating release.^8,17^

**Figure 2.**
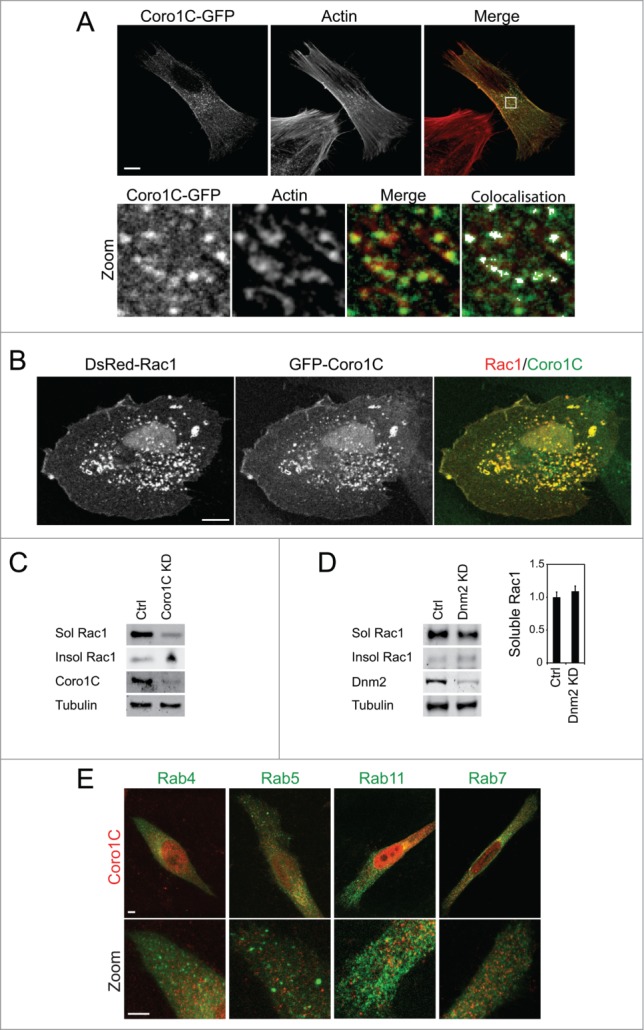
Coro1C trafficks Rac1 through actin-rich vesicles. (**A**) GFP-Coro1C-expressing fibroblasts were spread on fibronectin and fixed and stained with phalloidin. Co-localization highlighter analysis indicates the presence of Coro1C-positive and actin-positive vesicles. Bar = 10 μm. (**B**) Fibroblasts coexpressing GFP-Coro1C and DsRed-Rac1 were spread on fibronectin and fixed. Bar = 10 μm. (**C and D**) Weak detergent lysates of control, coronin-1C-knockdown and dynamin (Dnm2)-knockdown fibroblasts were fractionated by centrifugation and blotted for distribution of Rac1 between soluble and insoluble fractions analyzed by Western blot. n = 4, error bars indicate standard error. (**E**) HeLa cells stably expressing the appropriate GFP-tagged Rab proteins were spread on fibronectin, fixed and stained for coronin-1C. Bar = 5 μm.

The interactions of coronin-1C with both Rac1 and Arp2/3 suggest a possibility of coordination between cytoskeletal regulators and the cytoskeleton itself, however the position of the Rac1-binding site raises questions about possible competition with actin binding. Actin binding by the coronin-1C propeller is mediated by Arg28, which is conserved between type I coronins (**Fig. 2D**, blue).^10^ Although the actin and Rac1-binding sites do not directly overlap, our structural model shows that proximity of the sites would prevent simultaneous binding of actin and Rac1 to the coronin-1C propeller. However, such competition would not necessarily preclude simultaneous association with actin and Rac1, as uniquely among coronins, coronin-1C contains a second actin-binding motif within the linker region of the tail domain.^10^

The relationship between binding sites caused us to examine the localization of coronin-1C, actin and Rac1 within the cell. It is noticeable that the previously reported accumulation of coronin-1C along the lateral edges of cells coincides with bundled actin stress fibers (2A), which is not typically where Rac1 would be expected to localize. GFP-coronin-1C was also detected in small vesicles in the perinuclear region of the cell, where it colocalised with actin puncta (**Fig. 2A**, zoom). This distribution agreed with previous observations that coronins colocalise with filamentous actin, and that actin-binding is central to the function of coronins.^9,18^ Although endogenous Rac1 is diffusely distributed in the cell, coronin-1C/Rac1 positive puncta can be observed, and when Rac1 and coronin-1C are overexpressed, numerous, large costained vesicles are formed (**Fig. 2B**). Formation of these vesicles is consistent with our recent report that knockdown of coronin-1C caused Rac1 to become trapped in the detergent-insoluble membrane at the sides of the cell,^8^ indicating that coronin-1C plays a role in Rac1 trafficking. The immobilization of Rac1 was demonstrated to be dependent on membrane-association, rather that association with the actin cytoskeleton, as diffusion of a CAAX box-mutant Rac1, which is unable to associate with the cell membrane, was unaffected by knockdown of coronin-1C.

The model of coronin-1C-mediated Rac1 trafficking led us to examine the role of canonical endocytic and trafficking molecules in the process. Dynamin is one of the major mediators of eukaryotic endocytosis, and is responsible for promoting fission of tubulating membrane into newly formed endocytic vesicles.^19^ To test release of Rac1 from insoluble membrane microdomains, cells were fractionated into detergent-soluble and detergent-insoluble fractions. In control cells, Rac1 was detected predominantly in detergent-soluble membrane, but shifted to the insoluble fraction upon knockdown of coronin-1C (**Fig. 2C**). By contrast, depletion of dynamin-2 (Dnm2, the dynamin isoform expressed in fibroblasts) did not lead to Rac1 being trapped in the detergent-insoluble fraction (**Fig. 2D**), demonstrating that coronin-1C-mediated release of Rac1 is dynamin independent. To further investigate whether coronin-1C directs Rac1 through a canonical trafficking pathway, we examined the localization of coronin-1C in cells stably expressing GFP-Rab4, -Rab5, -Rab11 and Rab-7 (**Fig. 2E**). Rab positive vesicles are less punctate than coronin-1C-stained vesicles, and there was no apparent overlap in localization. Collectively these experiments demonstrate that coronin-1C does not direct Rac1 through the dynamin-dependent, Rab-mediated pathway that is responsible for most endocytosis. The fact that the coronin-1C vesicles co-stain for actin might suggest some form of pinocytic mechanism and this is certainly an area for future investigation. It would also be worth examining whether coronin-1C associates with other trafficking mediators, such as members of the sorting nexin family of proteins, which direct endocytic cargos through a range of trafficking pathways.

**Figure 3.**
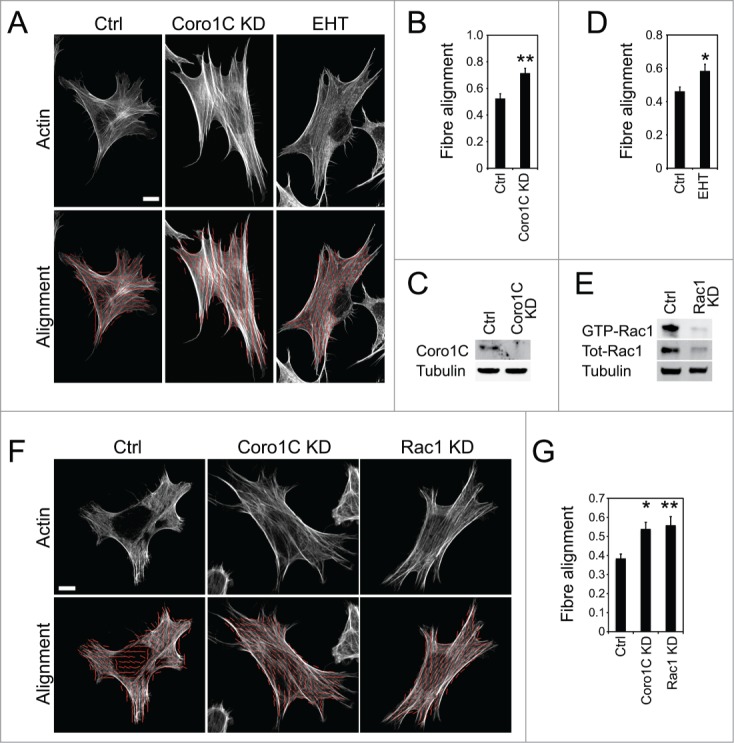
Inhibition of Coro1C or Rac1 increase stress fiber alignment. Fibroblasts spread on fibronectin, fixed and stained with phalloidin were analyzed for alignment of actin stress fibers . (**A–D**) Control, coronin-1C-kockdown or treated with the Rac1 inhibitor (EHT 1864) human fibroblast. (**E–G**) Control, coronin-1C-kockdown, or Rac1-knockdown MEFs. (**A and F**) Example images of actin staining. The images were segmented and ellipses fitted to the angle of maximum pixel intensity. The alignment of ellipses was then calculated as described in the methods, and average values plotted (**B**, **D**, **and G**). (**C and E**) Confirmation of knockdown by Western blot. n = 15. Error bars indicate standard error. Significance was tested by T-test, *P < 0.05, **P<0.005. Bar = 10 μm.

A key question is how coronin-1C affects organization of the actin cytoskeleton within the cell. Due to the interaction with the Arp2/3 complex, it would not be surprising if coronins affected actin filament dynamics, although the presence of multiple coronin isoforms introduces redundancy into the system.^9^ However, the unique Rac1 and RCC2-binding motifs of coronin-1C would cause us to expect a phenotype in depleted cells, and led us to compare the effects of depleting coronin-1C and inhibiting Rac1. Knockdown of coronin-1C did not affect the formation of actin stress fibers, but did appear to affect the alignment. To analyze the change in cytoskeletal architecture, we divided images into sections and used fast-fourier transformations to fit ellipses to the distribution of fluorescence intensity and measure the angle of maximum intensity in each section, so that the alignment of fibers between multiple sections could be calculated. Coronin-1C-depleted cells exhibited a significantly increased actin alignment compared to control (**Fig. 3A–C**). A similar increase in actin alignment was observed when Rac1 was pharmacologically inhibited by EHT 1864 (**Fig. 3A and D**) or depleted using siRNA (**Fig. 3E–G**). The similarity in effects on actin structure implies that deregulation of Rac1 in coronin-1C knockdown cells is the cause, rather than consequence of, cytoskeletal reorganization. If loss of Rac1 signaling were downstream of actin alignment, inhibition of Rac1 would not have the same effect. The change in alignment is important because, given the role of coronins in suppressing actin branches by inhibition of Arp2/3, one might expect fiber alignment to be reduced in coronin-1C-knockdown cells. However, the opposite was observed, suggesting the effect on Rac1 regulation dominates.

**Figure 4.**
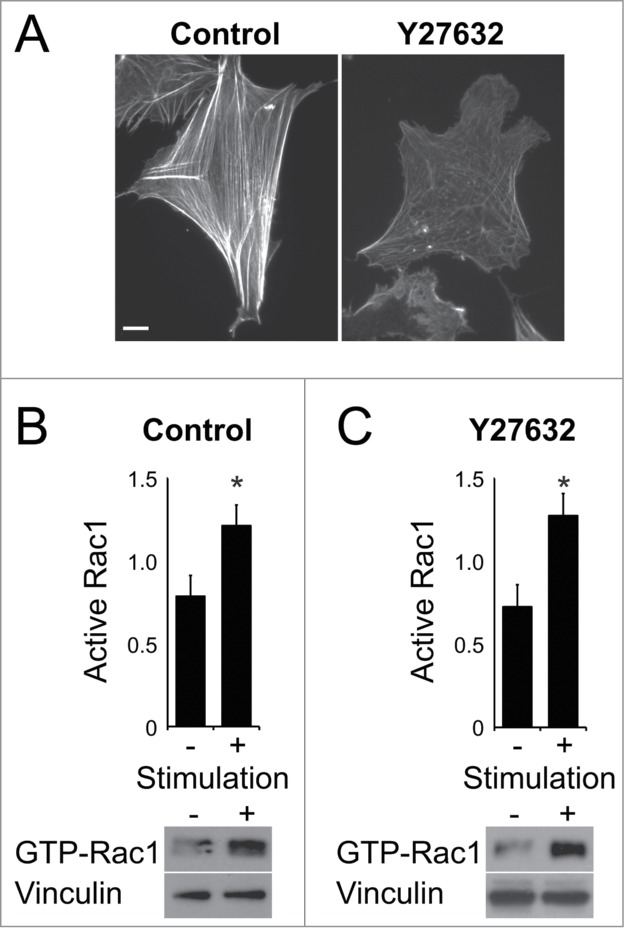
Rac1 activation is not dependent on actin stress fiber integrity. (**A**) Fibroblasts spread on fibronectin and treated with vehicle or Rho Kinase inhibitor (Y27632) were fixed and stained with phalloidin. Fibroblasts, spread on the integrin-binding domain of fibronectin and treated with vehicle (**B**) or Y27632 (**C**) inhibitor, were stimulated with the syndecan-4-binding fragment of fibronectin to trigger Rac1 activation. n = 4. Error bars indicate standard error. Significance was tested by T-test, *P < 0.05. Bar = 10 μm.

To test the hypothesis that Rac1 regulation lies upstream of actin alignment signals, we investigated the effect of cytoskeletal integrity on Rac1 activation. Actin stress fibers can be disassembled by treatment of cells with the Rho-kinase inhibitor, Y27632 (**Fig. 4A**). In control cells plated on a minimal integrin-binding ligand in the absence of serum, Rac1 could be activated by stimulation with a soluble syndecan-4-binding fragment of fibronectin, thus triggering adhesion-dependent signaling by engaging both fibronectin receptors (**Fig. 4B**). Importantly, activation of Rac1 was not dependent on the integrity of stress fibers, as cells treated with the Rho kinase inhibitor Y27632 still activated Rac1 in response to fibronectin (**Fig. 4C**). This observation does not rule out the involvement of all actin structures in coronin-1C function, as actin filaments, actin ruffles and actin vesicles were still observed in cells treated with Y27632 (**Fig. 4A**). Indeed, the fact that actin-associated vesicles still form may be critical to coronin-1C-dependent Rac1 trafficking. However it does demonstrate that the effects we report on Rac1 regulation in coronin-1C depleted cells are not simply a consequence of a reduction in actin crosslinking, and suggests that the originally described role of coronins in actin crosslinking, and the unique role of coronin-1C in redistributing Rac1 for reactivation are separate. However, the roles of coronin-1C in both regulation of actin branching by Arp2/3 and Rac1 redistribution, which itself regulates Arp2/3 through the WAVE complex, means that further investigation in this area is needed. The roles of Arp2/3 in lamellipodial extension and migration guidance,^20^ combined with the importance of intermediate Rac1 activity for persistent migration^21^ would make such investigations fruitful.

**Figure 5.**
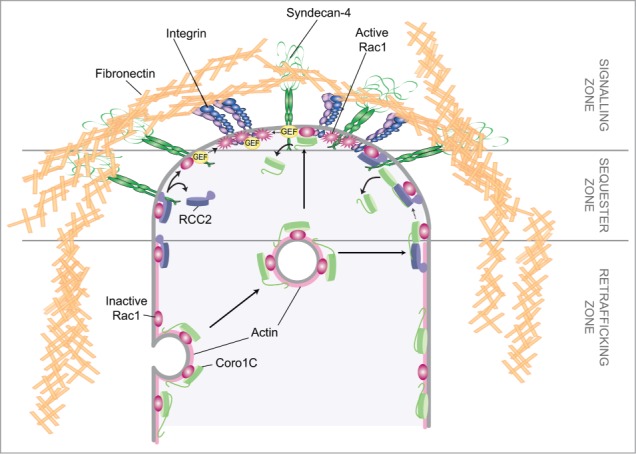
Coronin-1C-mediated Rac1 trafficking. Schematic of the pathway by which actin-associated coronin-1C redistribute Rac1 from the sides of the cell to the leading edge so that Rac1 can be activated at dense signaling clusters.

Other questions surrounding the process of coronin-1C-dependent Rac1 trafficking are the mechanisms by which Rac1 is released for activation and signaling. We have reported that coronin-1C and the sequestering molecule, RCC2, compete for the same binding site on Rac1 and that the RCC2 interaction has higher affinity.^8^ Indeed, it was found that trafficking of Rac1 from RCC2-containing protrusive membranes was unaffected by coronin-1C knockdown, and that only at lateral membranes was Rac1 mobility affected by coronin-1C expression. As coronin-1C is found throughout the plasma membrane as well as actin-associated vesicles, it is probable that coronin-1C mediated release is directional, and is responsible for movement of Rac1 from lateral to protrusive membrane (**Fig. 5**). Such a model is complemented by the poor affinity of coronin-1C for GTP-bound Rac1, which would cause movement of inactive Rac1 from lateral membranes, but not active Rac1 from protrusions. This hypothesis was supported by the observation that tagged Rac1 redistributed from lateral to protrusive membrane in control cells, but failed to do so upon knockdown of coronin-1C. We also reported that RCC2 could be out-competed by high concentrations of active guanine exchange factor (GEF). It follows from these results that local concentrations of RCC2, Coro1C and GEFs are important in determining areas of local Rac1 activation.

In our previous manuscript, we used the well-characterized Rac1-GEF Trio to model the competition between RCC2 and a GEF. However, the actual GEF responsible for this process in normal physiology is unknown, and remains a prominent question moving this research forward. In addition, the factors which determine relative concentrations of exchange factors at different regions of the membrane remain poorly characterized. A study by Goryachev and Pokhilko^22^ acknowledged the importance of maintaining appropriate concentrations of GTPase regulators at the cell membrane, and the likelihood that efficient GDP-GTP cycling can only take place within dense protein complexes. It is tempting to hypothesize that certain areas of the cell membrane are predisposed toward activation, although this study did not examine the role of additional matrix receptors in determining dynamics of Rac1 activation. For example, it may be that one fibronectin receptor, α5β1 integrin, ‘primes’ the focal adhesion for activation of Rac1 by a second fibronectin receptor, syndecan-4 (**Fig. 5**). If this is the case, then understanding the complexes formed between transmembrane receptors and the activation, sequestration and trafficking regulators of small GTPases will be the key to understanding migration guidance.

## Methods

### Cell culture and RNAi knockdown

The generation of immortalized MEFs has been described previously.^5^ To allow expression of the large T antigen, MEFs were cultured at 33°C in DME, 10% FBS, 4.5 g/l glucose, 2 mM L‑glutamine and 20 U/ml IFNγ (Sigma, I4777). Telomerase-immortalized human fibroblasts were cultured at 37°C in DME, 15% foetal bovine serum, 4.5 g/l glucose, 25 mM HEPES and 2 mM L‑glutamine. Where appropriate, fibroblasts were transfected with pDsRed-Rac1 and GFP-Coro1C using Fugene HD (Promega, E2311). HeLa cells stably expressing GFP-Rab4, Rab5, Rab7 or Rab11 were a gift from Professor Pete Cullen, University of Bristol, and were cultured at 37°C in DME, 10% foetal bovine serum, 4.5 g/l glucose and 2 mM L‑glutamine.

siRNA duplexes with ON TARGET^TM^ modification for enhanced specificity were purchased from Dharmacon (Thermo Fisher Scientific). Sequences targeted the sense strand of mouse Coro1C (CCGUUGAAUUAAUUACGUA) or Rac1 (AGACGGAGCUGUUGGUAAAUU), or human Coro1C (GCACAAGACUGGUCGAAUU). For knockdown, 160 pmol of targeted or control oligo was transfected into a 90% confluent 25-cm^2^ flask using Dharmafect2 reagent (Dharmacon, T-2002-03). After 18 hours, the cells were passaged and cultured for 2 days before transfecting again to ensure substantial knockdown. Cells were passaged 18 hours after the second round of transfection and used within 2-3 days. Expression of target proteins in comparison with mock-transfected cells was tested by Western blotting.

### Immunofluorescence

13-mm diameter glass coverslips were coated with 10 μg/ml fibronectin (Sigma, F1141) in Dulbecco's PBS containing calcium and magnesium (Sigma, D8662), and blocked with 10 mg/ml heat-denatured BSA for 30 minutes at room temperature. Cells were spread for 4 hours at 1.25 × 10^4^ cells per coverslip in DME, 10% FBS, 4.5 g/l glucose, 25 mM HEPES, and then treated with 50 μM EHT1864 if appropriate. Spread cells were fixed with 4% (w/v) paraformaldehyde, permeabilized with 0.5% (w/v) TrX diluted in PBS, and blocked with 3% (w/v) BSA in PBS. Fixed cells were stained with phalloidin (Sigma, 65906), mounted in Prolong®Antifade (Invitrogen, P36934) and photographed on a Leica SP5-II confocal laser scanning microscope using a 100x, NA 1.4 PlanApo objective. Maximum projection images were compiled, bandpass filtered, and analyzed using ImageJ software.

## Fiber Alignment Analysis

Alignment is measured using the dot product. Each straight segment of the track defines a vector $v_{i}$, which may be compared to all the other segments of the track. The value of the dot product $d_{ij}$ between vectors $v_{i}$ and $v_{j}$ is

$$d_{ij}=\frac{v_{i}•v_{j}}{\left|v_{i}||v_{j}\right|}$$

If the vectors are parallel $d_{ij}=1$, perpendicular gives $d_{ij}=0$ and reversed gives $d_{ij}=-1$. The value of $d_{ij}$ is then averaged over all segments, and the operation is in turn repeated for all N segments in the track to give

$$d=\frac{\sum_{i=1}^{i=N}\frac{\sum_{j=1}^{j=N}d_{ij}}{N}}{N}$$

*d* is then scaled so that for random arrangement of fibers, *alignment* = 0, for parallel fibers, *alignment* = 1

alignment=2(d−0.5)

### Homology modeling and docking studies

The Coro1C homology model was generated using the ESyPred3D server (Lambert et al., 2002) (http://www.fundp.ac.be/urdm/bioinf/esypred/), followed by model building in MODELLER and macromolecular docking with the Rac1-GDP crystal structure (Tarricone et al., 2001) (PDB 1I4D) using the ClusPro sever (Comeau et al., 2004) (http://cluspro.bu.edu/home.php).

### Cell fractionation and Rac1 activity

Tissue culture-treated plastic dishes (Corning BV, 430167) were coated with 20 μg/ml recombinant fibronectin polypeptide encompassing type III repeats 6‑10 that comprises the a_5_b_1_-integrin ligand (50K).^5^ To prevent *de novo* synthesis of ECM and other syndecan-4 ligands, MEFs were pretreated with 25 μg/ml cycloheximide (Sigma, C7698) for 2 hours and spread for 2 hours in DME, 4.5 g/l glucose, 25 mM HEPES, 25 μg/ml cycloheximide. Where appropriate, spread cells were treated 10 μM Rho Kinase inhibitor, Y27632 (Millipore, 688000) or 10 μg/ml recombinant fibronectin polypeptide encompassing type III repeats 12-14 that comprises the syndecan-4 ligand (H/0).^5^ For cell fractionation, detergent-resistant membrane and cytoskeletal components were separated by cetrifugation following lysis with 20 mM HEPES (pH 7.4), 10% (v/v) glycerol, 140 mM NaCl, 1% (v/v) Nonidet P-40, 4 mM EGTA, 4 mM EDTA. For GTPase signaling assays, active Rac1 was precipitated from cell lysates using GST-PAK-CRIB as bait.

## References

[1] Vicente-Manzanares M, Webb DJ, Horwitz AR. Cell migration at a glance. J Cell Sci 2005; 118:4917-9; PMID:16254237; http://dx.doi.org/10.1242/jcs.02662 16254237

[2] Aman A, Piotrowski T. Cell migration during morphogenesis. Dev Biol 2010; 341:20-33; PMID:19914236; http://dx.doi.org/10.1016/j.ydbio.2009.11.014 19914236

[3] Swaney KF, Huang CH, Devreotes PN. Eukaryotic chemotaxis: a network of signaling pathways controls motility, directional sensing, and polarity. Annu Rev Biophys 2010; 39:265-89; PMID:20192768; http://dx.doi.org/10.1146/annurev.biophys.093008.131228 20192768 PMC4364543

[4] Le Clainche C, Carlier MF. Regulation of actin assembly associated with protrusion and adhesion in cell migration. Physiol Rev 2008; 88:489-513; PMID:18391171; http://dx.doi.org/10.1152/physrev.00021.2007 18391171

[5] Bass MD, Roach KA, Morgan MR, Mostafavi-Pour Z, Schoen T, Muramatsu T, Mayer U, Ballestrem C, Spatz JP, Humphries MJ. Syndecan-4-dependent Rac1 regulation determines directional migration in response to the extracellular matrix. J Cell Biol 2007; 177:527-38; PMID:17485492; http://dx.doi.org/10.1083/jcb.200610076 17485492 PMC1885470

[6] Echtermeyer F, Streit M, Wilcox-Adelman S, Saoncella S, Denhez F, Detmar M, Goetinck P. Delayed wound repair and impaired angiogenesis in mice lacking syndecan-4. J Clin Invest 2001; 107:R9-R14; PMID:11160142; http://dx.doi.org/10.1172/JCI10559 11160142 PMC199172

[7] Liu S, Kapoor M, Leask A. Rac1 expression by fibroblasts is required for tissue repair in vivo. Am J Pathol 2009; 174:1847-56; PMID:19349358; http://dx.doi.org/10.2353/ajpath.2009.080779 19349358 PMC2671273

[8] Williamson RC, Cowell CA, Hammond CL, Bergen DJ, Roper JA, Feng Y, Rendall TC, Race PR, Bass MD. Coronin-1C and RCC2 guide mesenchymal migration by trafficking Rac1 and controlling GEF exposure. J Cell Sci 2014; 127:4292-307; PMID:25074804; http://dx.doi.org/10.1242/jcs.154864 25074804 PMC4179493

[9] Chan KT, Creed SJ, Bear JE. Unraveling the enigma: progress towards understanding the coronin family of actin regulators. Trends Cell Biol 2011; 21:481-8; PMID:21632254; http://dx.doi.org/10.1016/j.tcb.2011.04.004 21632254 PMC3163407

[10] Chan KT, Roadcap DW, Holoweckyj N, Bear JE Coronin 1C harbors a second actin binding site that confers cooperative binding to F-actin. Biochem J 2012; 2012:27. 10.1042/BJ20120209 PMC3339874 22364218

[11] Spoerl Z, Stumpf M, Noegel AA, Hasse A. Oligomerization, F-actin interaction, and membrane association of the ubiquitous mammalian coronin 3 are mediated by its carboxyl terminus. J Biol Chem 2002; 277:48858-67. Epub 2002 Oct 10; PMID:12377779; http://dx.doi.org/10.1074/jbc.M205136200 12377779

[12] Xavier CP, Rastetter RH, Blomacher M, Stumpf M, Himmel M, Morgan RO, Fernandez MP, Wang C, Osman A, Miyata Y, et al. Phosphorylation of CRN2 by CK2 regulates F-actin and Arp2/3 interaction and inhibits cell migration. Sci Rep 2012; 2:241; PMID:22355754; http://dx.doi.org/10.1038/srep00241 22355754 PMC3268813

[13] Humphries JD, Byron A, Bass MD, Craig SE, Pinney JW, Knight D, Humphries MJ. Proteomic analysis of integrin-associated complexes identifies RCC2 as a dual regulator of Rac1 and Arf6. Sci Signal 2009; 2:ra51; PMID:19738201; http://dx.doi.org/10.1126/scisignal.2000396 19738201 PMC2857963

[14] Appleton BA, Wu P, Wiesmann C. The crystal structure of murine coronin-1: a regulator of actin cytoskeletal dynamics in lymphocytes. Structure 2006; 14:87-96; PMID:16407068; http://dx.doi.org/10.1016/j.str.2005.09.013 16407068

[15] Tarricone C, Xiao B, Justin N, Walker PA, Rittinger K, Gamblin SJ, Smerdon SJ. The structural basis of Arfaptin-mediated cross-talk between Rac and Arf signalling pathways. Nature 2001; 411:215-9; PMID:11346801; http://dx.doi.org/10.1038/35075620 11346801

[16] Swaminathan K, Muller-Taubenberger A, Faix J, Rivero F, Noegel AA. A Cdc42- and Rac-interactive binding (CRIB) domain mediates functions of coronin. Proc Natl Acad Sci U S A 2014; 111:E25-33; PMID:24347642; http://dx.doi.org/10.1073/pnas.1315368111 24347642 PMC3890859

[17] Castro-Castro A, Ojeda V, Barreira M, Sauzeau V, Navarro-Lerida I, Muriel O, Couceiro JR, Pimentel-Muíños FX, Del Pozo MA, Bustelo XR. Coronin 1A promotes a cytoskeletal-based feedback loop that facilitates Rac1 translocation and activation. Embo J 2011; 30:3913-27; PMID:21873980; http://dx.doi.org/10.1038/emboj.2011.310 21873980 PMC3209784

[18] Cai L, Makhov AM, Bear JE. F-actin binding is essential for coronin 1B function in vivo. J Cell Sci 2007; 120:1779-90; PMID:17456547; http://dx.doi.org/10.1242/jcs.007641 17456547

[19] Ferguson SM, De Camilli P. Dynamin, a membrane-remodelling GTPase. Nat Rev Mol Cell Biol 2012; 13:75-88; PMID:22233676; http://dx.doi.org/10.1038/nrm3266 22233676 10.1038/nrm3266 PMC3519936

[20] Suraneni P, Rubinstein B, Unruh JR, Durnin M, Hanein D, Li R. The Arp2/3 complex is required for lamellipodia extension and directional fibroblast cell migration. J Cell Biol 2012; 197:239-51; PMID:22492726; http://dx.doi.org/10.1083/jcb.201112113 22492726 PMC3328382

[21] Pankov R, Endo Y, Even-Ram S, Araki M, Clark K, Cukierman E, Matsumoto K, Yamada KM. A Rac switch regulates random versus directionally persistent cell migration. J Cell Biol 2005; 170:793-802; PMID:16129786; http://dx.doi.org/10.1083/jcb.200503152 16129786 PMC2171343

[22] Goryachev AB, Pokhilko AV. Computational model explains high activity and rapid cycling of Rho GTPases within protein complexes. PLoS Comput Biol 2006; 2:e172; PMID:17140284 17140284 10.1371/journal.pcbi.0020172 PMC1676031

